# Functional Brain Dysconnectivity in Parkinson's Disease: A 5‐Year Longitudinal Study

**DOI:** 10.1002/mds.29026

**Published:** 2022-04-14

**Authors:** Sahar Yassine, Ute Gschwandtner, Manon Auffret, Sophie Achard, Marc Verin, Peter Fuhr, Mahmoud Hassan

**Affiliations:** ^1^ Univ Rennes, Inserm LTSI ‐ U1099 Rennes F‐35000 France; ^2^ NeuroKyma Rennes F‐35000 France; ^3^ Department of Neurology Hospitals of the University of Basel Basel Switzerland; ^4^ Comportement et noyaux gris centraux, EA 4712, CHU Rennes Rennes France; ^5^ University Grenoble Alpes CNRS, Inria, Grenoble INP LJK 38000 Grenoble France; ^6^ Movement Disorders Unit, Neurology Department Pontchaillou University Hospital Rennes France; ^7^ Institut des Neurosciences Cliniques de Rennes (INCR) Rennes France; ^8^ MINDig Rennes F‐35000 France; ^9^ School of Science and Engineering Reykjavik University Reykjavik Iceland

**Keywords:** Parkinson's disease, functional brain networks, electroencephalography, follow‐up study, movement disorders

## Abstract

**Background:**

Tracking longitudinal functional brain dysconnectivity in Parkinson's disease (PD) is a key element to decoding the underlying physiopathology and understanding PD progression.

**Objectives:**

The objectives of this follow‐up study were to explore, for the first time, the longitudinal changes in the functional brain networks of PD patients over 5 years and to associate them with their cognitive performance and the lateralization of motor symptoms.

**Methods:**

We used a 5‐year longitudinal cohort of PD patients (n = 35) who completed motor and non‐motor assessments and sequent resting state (RS) high‐density electroencephalography (HD‐EEG) recordings at three timepoints: baseline (BL), 3 years follow‐up (3YFU) and 5 years follow‐up (5YFU). We assessed disruptions in frequency‐dependent functional networks over the course of the disease and explored their relation to clinical symptomatology.

**Results:**

In contrast with HC (n = 32), PD patients showed a gradual connectivity impairment in α2 (10‐13 Hz) and β (13–30 Hz) frequency bands. The deterioration in the global cognitive assessment was strongly correlated with the disconnected networks. These disconnected networks were also associated with the lateralization of motor symptoms, revealing a dominance of the right hemisphere in terms of impaired connections in the left‐affected PD patients in contrast to dominance of the left hemisphere in the right‐affected PD patients.

**Conclusions:**

Taken together, our findings suggest that with disease progression, dysconnectivity in the brain networks in PD can reflect the deterioration of global cognitive deficits and the lateralization of motor symptoms. RS HD‐EEG may be an early biomarker of PD motor and non‐motor progression. © 2022 The Authors. *Movement Disorders* published by Wiley Periodicals LLC on behalf of International Parkinson and Movement Disorder Society

## Graphical abstract

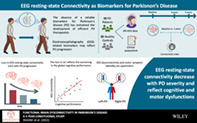

## Introduction

Parkinson's disease (PD) is one of the most frequent neurodegenerative disorders in the elderly population.^1^ Along with its well‐known prominent motor symptoms (resting tremor, bradykinesia, rigidity, and postural imbalance), a broad spectrum of non‐motor disturbances (cognitive impairment up to dementia, neuropsychiatric disturbances, autonomic or sleep disorders, etc.) can manifest from early (even prodromal) disease stages onward diminishing the quality of life of the patients.^2^, ^3^, ^4^ Pathologically, the most prevalent hypotheses suggest that the degeneration of dopaminergic neurons in the nigrostriatal system associated with Lewy body inclusions are the leading features in most forms of PD at early stages.^5^, ^6^ However, with the disease progression, the neuropathological process may propagate across interconnected networks reaching the cortex and causing functional alterations within and between brain regions.^7^ Still, these neuropathological insights do not sufficiently expound the heterogeneous phenotype^8^, ^9^ of the patients and the progression‐related changes in their brain activity.

Functional disruptions related to both motor and cognitive deficits at early and advanced PD stages were reported using mainly functional magnetic resonance imaging techniques (fMRI).^10^, ^11^, ^12^, ^13^ Moreover, electro/magneto‐encephalography (EEG/MEG) are increasingly shown as powerful, cost‐effective, and non‐invasive electrophysiological techniques, to explore functional brain networks in neurodegenerative disorders.^14^, ^15^, ^16^, ^17^, ^18^ In PD, MEG/EEG studies uncovered an increase in the cortico‐cortical connectivity, characterizing PD patients from earliest disease clinical stages onward,^19^, ^20^ whereas decreases in the functional connectivity, mainly in the frontotemporal networks, were proclaimed to reflect the development of mild cognitive impairment and dementia conditions in advanced stages.^17^, ^21^, ^22^ Reductions in the weight of the functional connections associated with the cognitive phenotype of PD patients were also revealed in several EEG studies.^23^, ^24^ However, an important limitation in these findings lies in the fact that these studies were all achieved in a single time point rather than longitudinally, a key point to ultimately develop biomarkers in PD.

Here, we used resting state high density (HD)‐EEG recordings of 77 PD patients that underwent a follow‐up study at baseline (BL), 3‐year follow‐up (3YFU) and 5‐yearfollow‐up (5YFU) at the Basel university hospital. Our primary goal was to explore the disruptions in the functional brain networks of those PD patients between the BL, 3YFU, and the 5YFU. We examined their progressive evolution and compared it to that of age‐matched healthy control subjects. In addition, we correlated the patient‐specific altered network with the change in the clinical scores. Finally, to link the disrupted networks with the lateralized motor symptoms of the patients, we revealed different altered networks between BL and 5YFU characterizing the progression of the disease in the left‐affected (LPD) and right‐affected (RPD) patients.

## Materials and Methods

### Participants

Patients were selected from a longitudinal study cohort of patients with idiopathic PD and healthy controls (HC). Patients were recruited from the outpatient clinic of the Department of Neurology and Neurophysiology of the Hospital of the University of Basel (City of Basel, Switzerland) in the period from 2011 to 2015 based on the following selection criteria: PD according to United Kingdom Parkinson's Disease Society Brain Bank criteria,^25^ Mini‐Mental State Examination (MMSE) above 24/30, no history of vascular and/or demyelinating brain pathology, and sufficient knowledge of the German language. HC were matched in age and education. Included patients underwent neurological, cognitive, and EEG examinations at BL and follow‐ups after a mean interval of 3 years and 5 years. The study was approved by the local ethics committees (Ethikkommission beider Basel, Basel; Switzerland; EK 74/09) and all patients gave written informed consent before the study inclusion. Specialists who performed the assessment of the patients were unaware of the details of this study. The main cohort included 77 patients at BL, from which 42 did not complete all the follow‐ups examinations (see Table S1 for the detailed demographic and clinical measures of the main cohort). All patients were under dopaminergic medications during the examinations. To be noted, 10 patients used cholinesterase inhibitors. We first analyzed the subgroup of 35 PD patients who underwent all the examinations at BL, 3YFU, and 5YFU, and we then cross‐validated the results on the remaining patients. The HC group included 32 participants at BL, from which 21 completed the 3YFU and 3 the 5YFU (see Fig. S1 for details of the subject's study flow). The 35 PD patients were also classified into two subgroups by a specialist based on the dominant side of their motor symptoms in all the three visits: LPD patients (n = 23) and RPD patients (n = 10). The division was done using the lateralized items of the Unified Parkinson's Disease Rating Scale III (UPDRS‐III) score (items 20–26). Patients with symmetric symptoms in all visits (n = 2) were excluded from this subgroup division.

### Data Acquisition and Preprocessing

Resting state EEG data were recorded in the afternoons using a 256‐channel EEG System (Netstation 300, EGI, Eugene, OR) during continuous 12 minutes. Patients were seated comfortably in a relaxing chair, instructed to close their eyes and relax while staying awake with minimum eye and body movements. An EEG technician present in the recording room controlled for vigilance of the patients. The sampling rate of the signals was set to 1000 Hz. The high density (HD‐EEG) signals were segmented into epochs of 40 seconds each and the first epoch from each recording was discarded from the study. They were preprocessed automatically using the open‐source toolbox Automagic^26^ and the first six epochs with best quality metrics were retained for the rest of the analysis. See Appendix S1, method section, for more technical details about the preprocessing.

### Brain Networks Reconstruction

After preprocessing, the functional brain networks were estimated using the EEG source‐connectivity method.^27^ First the dynamics of cortical brain sources were reconstructed by solving the inverse problem. To do so, EEG channels and magnetic resonance imaging (MRI) template (ICBM152) were co‐registered, and using the OpenMEEG toolbox,^28^ a realistic head model was built. The weighted minimum norm estimate (wMNE)^29^ was used to calculate the regional time series of the 210 cortical regions of interest (ROIs) of the Brainnetome atlas.^30^ Afterward, the regional time series were filtered in different EEG frequency bands: θ (4–8 Hz), α1 (8–10 Hz), α2 (10–13 Hz), and β (13–30 Hz). For each frequency band, functional connectivity was computed between the reconstructed sources using the phase locking value (PLV).^31^ We obtained for each participant six dynamic connectivity matrices in each frequency band. Those matrices were ultimately averaged across time and epochs to obtain a single static functional connectivity matrix per frequency band, used in the further analysis.

### Statistical Analysis

The network‐based statistic (NBS)^32^ was used to identify the brain networks of PD patients that are significantly different between BL and 5YFU visits. This approach assumes that subnetworks of disrupted connections are more likely to indicate real alterations than isolated disconnections and has been shown to provide considerably greater statistical power than generic methods. Age, gender, education, levodopa equivalent daily dose (LEDD), and dominant side of motor symptoms were considered as confounding factors in this analysis. More details about the statistical analysis are provided in the Appendix S1, methods section.

### Network Index and Correlation Analysis

We defined a metric called network index (NI), inspired from previous work of Hassan et al,^23^ as the average weight (connectivity) of the output significant networks issued from NBS:

$$\mathrm{NI}=\frac{\sum_{i}^{N}W_{i}}{N},$$

where $W_{i}$ represents the connectivity value of the edge i of the significant network from NBS and N represents the total number of edges in this network. This NI was computed for each patient in each of the three visits to quantify the longitudinal disruptions in the networks as the disease progressed in time. For the correlation analysis, the Pearson's correlation was computed to evaluate the relationship between the NI and the clinical scores of PD patients at and between different timepoints. All parameters are described in the shared GitHub containing the codes necessary to reproduce the results (https://github.com/yassinesahar/FuncDysconnectivityPD).

## Results

### Participant Characteristics

Table 1 summarized the demographic and clinical characteristics of the PD patients and HC subjects included in this study. Their complete neuropsychological and neuropsychiatric assessments are shown in Table S2. There was no significance difference in age, sex, or duration of formal education between the PD patients and the HC at BL.

**TABLE 1:** Demographic and clinical characteristics of the participants at BL, 3YFU, and 5YFU visits

	Baseline			3YFU			5YFU	
	PD (n = 35)	HC (n = 32)	*P* value	PD (n = 35)	HC (n = 21)	*P* value	PD (n = 35)	HC (n = 3)
Sex (M/F)	26/9	18/14	0.13	—	10/11	—	—	2/1
Age (y)	67.4 (8.2)	65.3 (5.6)	0.24	70.4 (8.2)	68.7 (5.5)	0.37	72.5 (8.2)	65.7 (4.2)
Education (y)	15.2 (3.2)	13.8 (2.9)	0.07	—	13.6 (3)	—	—	11 (2)
Disease dur. (y)	4.1 (3.7)	NA	—	7.1 (3.7)	NA	—	9.2 (3.8)	NA
LEDD (mg/day)	555 (430)	NA	—	660 (449)	NA	—	647 (396)	NA
UPDRS‐III	13.9 (10.2)	NA	—	18.4 (9.7)	NA	—	18 (12.4)	NA
MoCA	26.2 (2.4)	26.8 (2.5)	0.26	25.1 (3.8)	27.3 (2.1)	0.016*	24.5 (5.5)	23.3 (4.6)

Values are expressed as mean (standard deviation). *Indicates results are significant.

BL, baseline; 3YFU, 3 year follow‐up; 5YFU, 5 year follow‐up; PD, Parkinson's disease patients; HC, healthy controls; M/F, male/female; y, years; LEDD, Levodopa equivalent daily dose; UPDRS‐III, Unified Parkinson's Disease Rating Scales motor ratings; MoCA, Montreal Cognitive Assessment, NA, Not Applicable.

### Longitudinal Alterations in Functional Connectivity Networks of PD Patients

**FIG 1:**
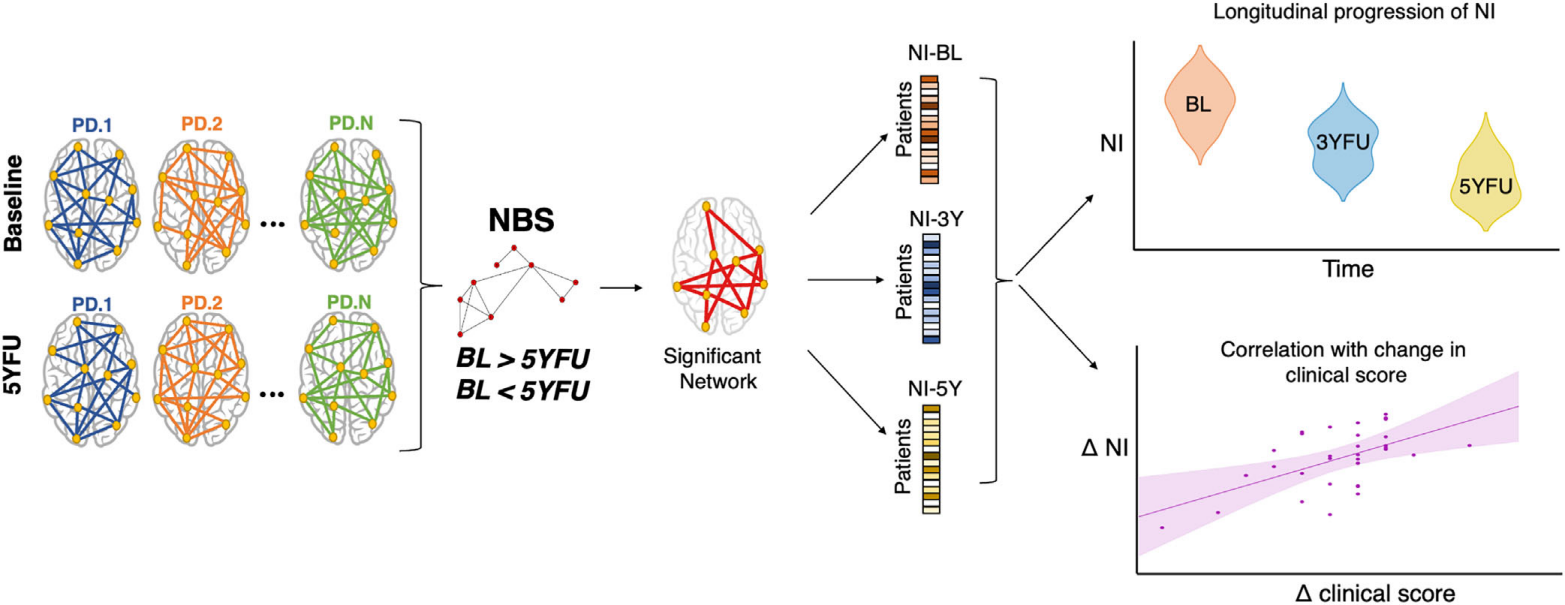
General description of the analysis. The connectivity networks of the PD patients at BL and 5YFU were compared using the Network‐Based Statistics (NBS) to retrieve a subnetwork of significantly hypo (BL > 5Y)/hyper (BL < 5Y) connectivity. From this significant network, A network index (NI) was attributed to each PD patient in each of the three visits to evaluate their progression in time and to correlate their longitudinal change with the change in clinical scores. [Color figure can be viewed at wileyonlinelibrary.com]

The overall pipeline of the study is summarized in Figure 1. We followed a data‐driven approach supposing that the networks at BL are different from those at 5YFU. The longitudinal change in the NI for both PD patients and HC was explored for the three timepoints. This includes the intermediate timepoint 3YFU whose networks were not used in the comparisons to confirm the overall tendency in the networks and to evaluate the potential correlations with the clinical scores.

### Decreasing Networks of PD Patients (BL > 5YFU)

**FIG 2:**
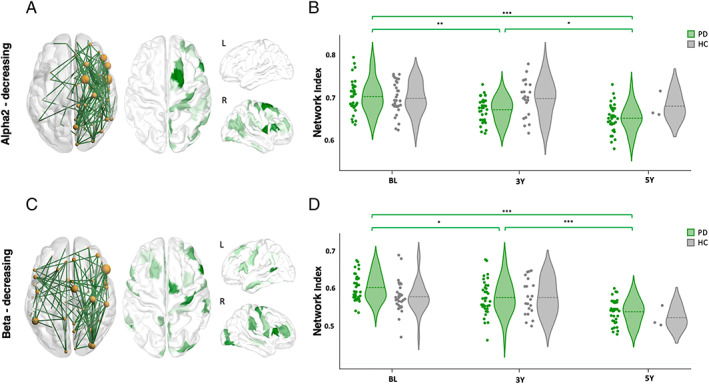
Dysconnectivity networks between BL and 5YFU and their corresponding highest degree regions in (**A**) α2, (**C**) β. Violin plot representing the longitudinal change of the network index of the PD patients and HC in (**B**) α2, (**D**) β. ***P < 0.001, **P < 0.01, *P < 0.05 (corrected for multiple comparisons using Bonferroni). [Color figure can be viewed at wileyonlinelibrary.com]

At the α2 band (10–13 Hz), results revealed a statistically significant network (*t* = 3.2, *P* = 0.021, corrected using permutation), where the connectivity at 5YFU was significantly lower than BL. This network included 125 connections and 72 regions located principally within the right hemisphere (88.8% of the edges and 88.9% of the regions). Regions with the highest number of connections (highest degree) were mainly part of the right superior and inferior frontal gyrus, right precentral gyrus and the right precuneus, with a dominance of the frontotemporal (12%), the frontofrontal (11.2%), and frontocentral (11.2%) connections (Fig. 2A). These results are in line with previous studies that linked the decrease in frontotemporal connectivity at the α band with the severity of disease progression.^17^, ^23^ The remarkable dominance of the right hemisphere will be explored later when conducting the analysis on the lateralized subgroups. Furthermore, to better understand the longitudinal changes in the network, the NI was computed for the three visits: BL, 3YFU, and 5YFU. Results showed that the NI undergoes a significant decrease between BL and 5YFU (*P* < 0.001). This decrease was progressive and significant between BL and 3YFU (*P* < 0.01) and likewise between 3YFU and 5YFU (*P* < 0.05). All *P* values were corrected using Bonferroni for multiple comparisons. This NI applied on the functional connectivity networks of the HC showed no significant longitudinal changes between BL and 3YFU (Fig. 2B).

Regarding the β band (13–30 Hz), the obtained network (*t* = 3.7, *P* = 0.009, corrected using permutation) comprised 103 connections issued from 58 regions. The right hemisphere was also dominant in terms of number of local edges (56.3%) and regions (58.6%). Highest degree regions were largely located in the right superior and inferior frontal, precentral and occipital cortex with a dominance of the frontotemporal (12.6%), fronto‐occipital (9.7%), and frontofrontal (8.7%) connections (Fig. 2C). A significant decrease in the NI was observed between BL and 5YFU (*P* < 0.001). This decrease was more severe in terms of linear slope between 3YFU and 5YFU (*P* < 0.001) than between BL and 3YFU (*P* < 0.05). As for the HC, they showed no significant change in their NI between BL and 3YFU, and because of their small sample size at 5YFU, we were not able to statistically test the decrease observed between 3YFU and 5YFU (Fig. 2D). Results of the other frequency bands, the increasing networks and the cross validation on the entire cohort are provided in the Appendix S1.

### Relationship between the NI and the Global Cognitive and Motor Scores

**FIG 3:**
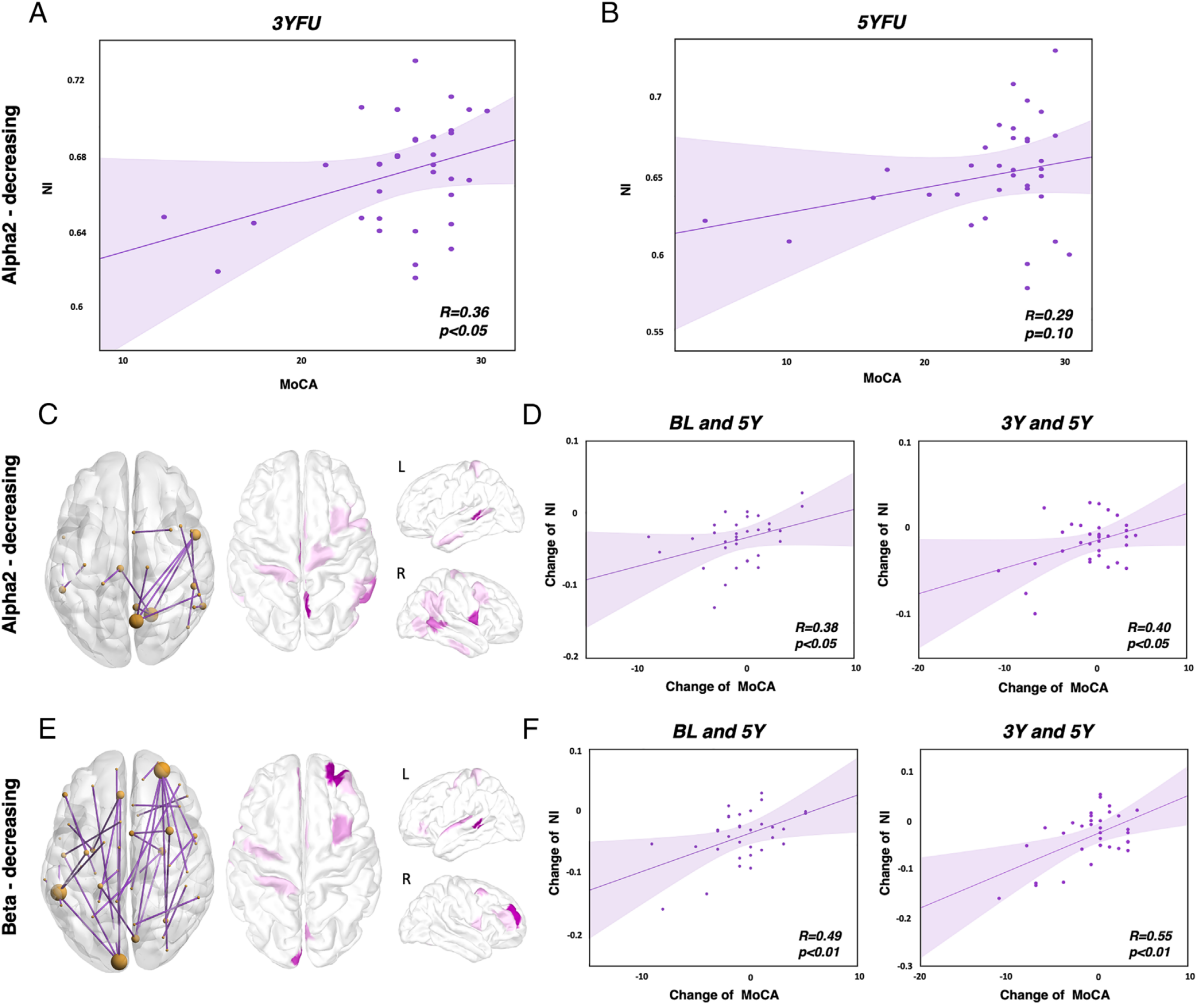
Longitudinal change in the NI and in the MoCA score of PD patients. Correlation between the NI (issued from the hypo‐connectivity networks reported above) and the MoCA score of PD patients at (**A**) 3YFU and (**B**) 5YFU. Dysconnectivity subnetworks and corresponding highest degree regions where the longitudinal change in the value of connectivity correlates with the longitudinal change in MoCA score of PD patients in (**C**) α2 and (**E**) β. Correlation between the change in NI and the change in MoCA observed between BL and 5YFU (left), 3YFU, and 5YFU (right) in (**D**) α2 and (**F**) β. [Color figure can be viewed at wileyonlinelibrary.com]

Results showed a global positive correlation between the NI of PD patients and their global cognitive score represented by the Montreal Cognitive Assessment score (MoCA) at both follow‐up timepoints with a statistical significance at the intermediate timepoint 3YFU (*r* = 0.36, *P* < 0.05) (Fig. 3A,B). In addition, we uncovered a subnetwork issued from the previously found dysconnectivity networks, in which the longitudinal changes in the connectivity of its edges correlates with the longitudinal changes in the MoCA scores between BL and 5YFU. In α2, a subnetwork was revealed comprising 16 connections located mostly in the right hemisphere and its corresponding NI was computed (Fig. 3C). A positive significant correlation was observed between the change in both NI and MoCA between BL and 5YFU (*r* = 0.64, *P* < 0.001). This positive correlation persists also when introducing the NI of the intermediate timepoint (3YFU) reaching a significant level mainly between 3YFU and 5YFU (*r* = 0.40, *P* < 0.05). Concerning the subnetwork of the β band, it involved 40 connections distributed between both hemispheres (Fig. 3E). The longitudinal change of its corresponding NI was highly correlated with the change in MoCA score between BL and 5YFU (*r* = 0.72, *P* < 0.001) as well as between 3YFU and 5YFU (*r* = 0.55, *P* < 0.01). To ensure that the significant correlation observed in the change between BL and 5Y in both bands was not driven by the 2 patients that showed a sharp decrease in their MoCA scores (as it might be shown in the Fig. S4), we computed the correlation after excluding these patients. Results show that the correlation remains significant in α2 (*r* = 0.38, *P* < 0.05) (Fig. 3D) and β (*r* = 0.49, *P* < 0.01) (Fig. 3F). We also found significant correlation between the changes in NI and the changes in motor score (the UPDRS‐III) at α2 (*r* = −0.38, *P* < 0.05) and β (*r* = −0.41, *P* < 0.05) between 3YFU and 5YFU. Figure and more details are provided in the Appendix S1.

### Disrupted Networks of PD Patients with Lateralized Motor Symptoms

The above obtained networks showed a majority of disrupted connections located in the right hemisphere in α2 and β bands. To investigate this asymmetry, we divided the PD patients into two subgroups according to the dominance of their lateralized motor symptoms: LPD and RPD. No significant difference in demographic and clinical tests was observed between both subgroups at baseline or within the same subgroup across the different visits (Table S3). To retrieve the specific dysconnectivity networks associated with the affected side of motor symptoms, we conducted the same previous analysis using NBS on the two lateralized subgroups of PD patients independently.

**FIG 4:**
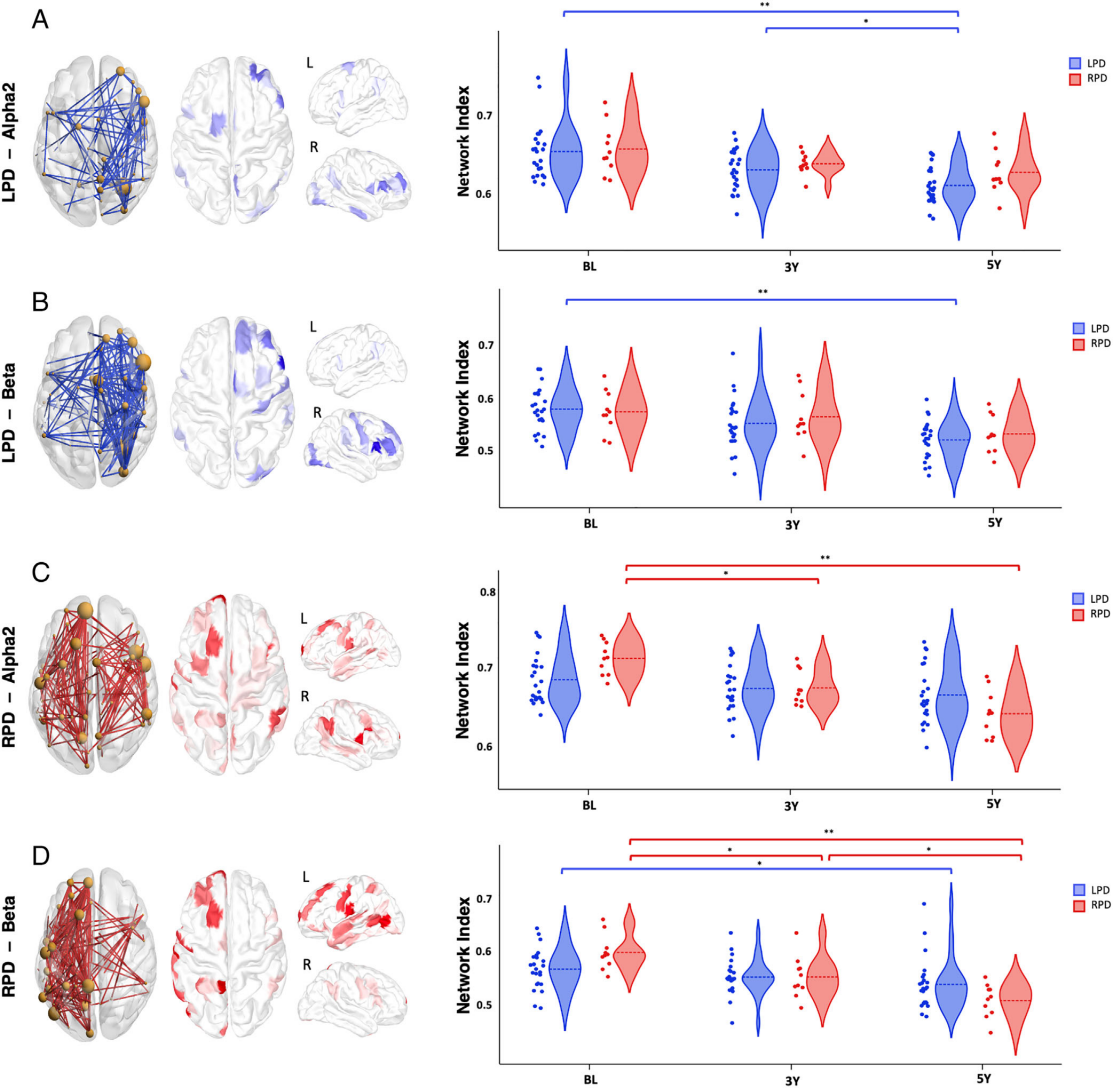
Dysconnectivity networks with their corresponding highest degree regions (left) and the longitudinal change of the NI in both LPD and RPD patients (right). (**A**) Network of the LPD patients in α2, (**B**) network of the LPD patients in β, (**C**) network of the RPD patients in α2, (**D**) network of the RPD patients in β. **P < 0.001, *P < 0.05 (corrected for multiple comparisons using Bonferroni). [Color figure can be viewed at wileyonlinelibrary.com]

For the LPD patients, the α2 band revealed a disrupted network (*t* = 3, *P* = 0.039, corrected) between BL and 5YFU comprising predominantly regions (71%) and connections (63%) within the right hemisphere. Highest degree regions were located principally in the frontal, occipital, and central lobes of the right hemisphere. The corresponding NI of those LPD patients showed a significant decrease between BL and 3YFU (*P* < 0.05), 3YFU and 5YFU (*P* < 0.05), BL and 5YFU (*P* < 0.001). However, this same NI does not reveal any significant differences between the three timepoints when applied on the RPD patients (Fig. 4A) and between BL and 3YFU when applied on the HC (*P* = 0.65). In contrast, dysconnectivity networks of the RPD patients between BL and 5YFU revealed a dominance of the left hemisphere in terms of altered connections. In α2 (*t* = 3.2, *P* = 0.044, corrected using permutation), the disrupted network encompassed a total of 167 connections and 83 regions. Although the majority of the edges (52.7%) were located within the left hemisphere with the highest degree regions among the left‐frontal and left‐central lobes, the right hemisphere comprises 40.1% of the disrupted edges mainly issued from regions in right‐central, right‐frontal, and right‐parietal lobes. The corresponding NI presented a significant decrease between BL and 3YFU (*P* < 0.05) and between BL and 5YFU (*P* < 0.001) in RPD patients. Yet, it did not mark any significant change between timepoints in LPD patients (Fig. 4C) and between BL and 3YFU in HC (*P* = 0.08). More details about these results in the βband are described in the Appendix S1.

## Discussion

In this longitudinal study, we investigated the evolution of the functional brain networks over a 5‐year period in PD patients using resting state HD‐EEG recordings. We unveiled disrupted networks in both α2 and β frequency bands where the functional connectivity decreases progressively between BL, 3YFU, and 5YFU, only in PD patients and not in HC. We also showed a positive correlation between the loss of functional connectivity in time and the deterioration in the MoCA score that is used largely to examine the global cognitive performance of PD patients. Furthermore, we noticed a dominance of the right hemisphere in terms of altered connections that led us to inspect more in depth the relationship between the motor symptoms lateralization of PD patients and the disruptions in their functional brain networks.

Our main findings revealed disrupted networks in α2 and β bands where the functional connectivity drops in function of time. Several previous cross‐sectional and longitudinal studies reported a decrease in the power of both alpha and beta bands in PD patients at early and advanced stages of the disease.^33^, ^34^, ^35^, ^36^ Likewise, these frequency bands were relevant in highlighting reductions in the functional connectivity associated with the motor and cognitive deteriorations over the course of the disease.^17^, ^22^, ^23^, ^37^ Our results are in line with these previous findings. In addition, our network index (computed at the patient‐level) derived from these networks has encountered a progressive decrease only in PD patients and not in healthy subjects in both bands, which could express the progression of the disease rather than the progression in age. Note that we obtained no significant differences when we performed the NBS analysis on PD patients between consecutive timepoints.

Regarding the spatial distribution of the disrupted connections and regions in α2 and β, the right hemisphere was predominant. In particular, the dominance of the right‐frontal and right‐central lobes was clear in both bands because they included most of the highest degree regions (involving most of the altered connections). A recent meta‐analysis showed that both right‐superior‐frontal and right‐central gyrus presented a reduced functional connectivity in cognitively impaired PD patients compared to HC.^38^ Besides, reductions in the functional connectivity of the right frontal and right somatomotor‐sensory cortex were previously reported to reflect reductions in cortical thinning in PD patients.^39^, ^40^ Moreover, we unfold here, that the frontotemporal connections were dominant in terms of altered connections in α2 and β. Indeed, functional disturbances between the frontal and temporal lobes in the α band were observed when comparing non‐demented to demented PD patients in several MEG study.^17^, ^22^ In EEG, these patterns of connections were relevant in α2 when comparing cognitively intact to cognitively impaired PD patients.^23^

We have also investigated the potential relationships between the lateralization of motor symptoms and the longitudinal change of the functional brain networks in PD patients. Although previous studies have reported changes in the cortical and subcortical areas related to the asymmetry of the motor symptoms^41^, ^42^ and linked them with the progression of both motor and cognitive symptoms of the disease,^43^, ^44^, ^45^ to date, no functional connectivity study has tackled their relationship longitudinally. When we conducted the same analysis based on NBS on the two subgroups separately, we found hypo‐connectivity networks in both α2 and β bands predominantly located in the right hemisphere and characterizing the longitudinal progression of the disease in the LPD patients in contrast to a dominance of the left hemisphere in the RPD patients. This contra‐laterality observed between the altered hemisphere and the side of the symptoms may be in line with previous studies that showed the correspondence between the side of the motor symptoms and the dopaminergic neuronal loss in the contralateral substantia nigra.^46^, ^47^ Furthermore, we found that the α2 frequency band revealed disturbances in the right hemisphere in RPD patients. Notably, when we first compared the evolution of the original NI over time in LPD and RPD patients, both subgroups showed significant decrease between different timepoints (Fig. S6). Therefore, alterations in the right hemisphere could be associated with the progression of the disease in both subgroups. Moreover, the previous results revealing the dominance of the right hemisphere in PD patients of the initial group could be related not only to the outnumber of the LPD patients over the RPD patients, (N=23 vs. N=10, respectively) but also to a pertinent pattern of disturbances within the right hemisphere that could reflect a common clinical deterioration in all patients. Another interpretation for these patterns of disturbances could be the fact that after more than 5 years of evolution, and despite the worsening of motor symptoms on the predominant side, the disease may become bilateral.^47^ This may also explain why the loss of connectivity in the left hemisphere characterizing the RPD patients in β band appears to be significant also in LPD patients (Fig. 4D).

In this study, we used the data of the PD patients that complete both follow‐up examinations to retrieve the disrupted networks in time, which relatively reduced the sample size from 77 to 35. However, we cross‐validated our results on the initial cohort to confirm the hypoconnectivity found between timepoints (Fig. S3 in Appendix S1). Unfortunately, having only three healthy subjects at 5YFU is not representative and prevents us from validating the longitudinal change in the network statistically in HC. We, therefore, only took into consideration the corresponding evolution occurring between BL and 3YFU. The relatively small sample size of RPD patients (n = 10) might also affect the statistical power of this comparison. As for the correlation analysis, we chose the global cognitive score MoCA (that covers several cognitive domains) rather than domain specific tests because our dysconnectivity analysis is more likely to reveal a large‐scale network rather than a domain‐specific one. For instance, the NI showed no correlation with tests that assessed only the visuospatial abilities. Finally, all patients were under dopaminergic treatments (*on* state) during both the EEG recordings and the neuropsychological assessments, which may mask the magnitude of their motor symptoms as well as their global cognitive performance and may, ultimately, affect our analysis. To overcome this issue, the affected side of PD patients was computed based on the lateralized items of the UPDRS‐III in the three timepoints, whereas the LEDD was considered as confounding in the correlation analysis. Despite these considerations, the effect of the dopaminergic medications could still be present in the measures of functional connectivity as shown by several studies.^48^, ^49^

In conclusion, the current study is the first to date to assess longitudinal changes in the functional brain networks of PD patients with the disease progression using HD‐EEG. Our findings suggest that disruptions in the networks may characterize not only the evolution of the disease, but also the evolution in the cognitive performance of the patients and the lateralization of their motor symptoms. Further investigations may lead to the identification of potential neuromarkers assisting in subtyping PD patients and predicting the evolution of their motor and cognitive symptoms in time.

## Financial Disclosures

None.

## Author Roles

(1) Research project: A. Conception, B. Organization, C. Execution; (2) Statistical Analysis: A. Design, B. Execution, C. Review and Critique; (3) Manuscript: A. Writing of First Draft, B. Review and Critique.

S.Y.: 1B, 1C, 2A, 2B, 3A

U.G: 1A, 1B, 2C, 3B

M.A.: 3B

S.A.: 2C, 3B

M. V: 1A, 2C, 3B

p.F.: 1A, 1B, 3B

M.H.: 1B, 1C, 2A, 2B, 3A

## Supporting information


**Appendix S1** Supporting information

## Data Availability

The data that support the findings of this study are available on request from the corresponding author. The data are not publicly available due to privacy or ethical restrictions.
