# Supplementary material for: Functional Brain Dysconnectivity in Parkinson's Disease: A 5‐Year Longitudinal Study
Source: Mov Disord. 2022 Apr 14;37(7):1444–53. doi: 10.1002/mds.29026 (PMC9543227; doi:10.1002/mds.29026)
Supplement: Supplementary file 1 — Appendix S1 Supporting information [file MDS-37-1444-s001.docx]

Supplementary materials

**Functional brain dysconnectivity in Parkinson’s disease: A 5-year longitudinal study**

Sahar Yassine^1,2^ MSc, Ute Gschwandtner^3^ MD, PhD, Manon Auffret^4^ PharmD, PhD, Sophie Achard^5^ PhD, Marc Verin^1,4,6,7^ MD, PhD, Peter Fuhr^3^ MD, PhD, Mahmoud Hassan^8,9^ PhD

^1^ Univ Rennes 1, LTSI - U1099, F-35000 Rennes, France

^2^ NeuroKyma, F-35000 Rennes, France

^3^ Dept. of Neurology, Hospitals of the University of Basel, Switzerland

^4^ Comportement et noyaux gris centraux, EA 4712, CHU Rennes, Univ Rennes 1, F-35000 Rennes, France

^5^ CNRS, Grenoble INP, GIPSA-Lab, University of Grenoble Alpes, Grenoble, France

^6^ Movement Disorders Unit, Neurology Department, Pontchaillou University Hospital, Rennes, France

^7^ Institut des Neurosciences Cliniques de Rennes (INCR), Rennes, France

^8^ MINDig, F-35000 Rennes, France

^9^ School of Science and Engineering, Reykjavik University, Reykjavik, Iceland

**Methods**

### Data acquisition and preprocessing

### Briefly, the Automagic toolbox is configured to filter the signals between 1 and 45 Hz, perform the electroencephalography (EOG) regression to eliminate the ocular artifacts (therefore the final number of channels is reduced to 239), and to detect and interpolate bad channels with high variance (higher than 20 μV) or whose amplitude exceeds the ±80 μV at any temporal point of the signal. The independent component analysis was also chosen in the configuration to remove further artefacts. Epochs whose Ratio of Bad Channels (RBC), one of Automagic quality metrics, exceeds the 0.15 threshold (representing 15% of the total number of electrodes) were excluded and the rest were sorted according the Overall High Amplitude (OHA) metric and only the first six epochs were chosen for the rest of the study. This step was confirmed manually by a visual inspection of the epochs and those with remaining artefacts were modified or removed totally from the study. All these parameters are described in the shared GitHub containing the codes necessary to reproduce the results (<https://github.com/yassinesahar/FuncDysconnectivityPD>) .

### Statistical Analysis

In brief, to identify the significant networks in NBS, a t-test was performed to evaluate the significant difference in the value of connectivity along the (N^2^-N)/2 = 21945 edges of the [210 x 210] connectivity matrices of the PD patients between the BL and 5YFU visits. A primary component-forming threshold (*p*<0.05) was then applied to identify a set of supra-threshold connections, within which all the possible connected components and their size could be determined. A corrected *p*-value was then evaluated for each component with respect to the null distribution of maximal connected component size obtained using a nonparametric permutation approach (2000 permutations). The analysis was performed with different thresholds for the t-test (range from 2.6 to 3.7) which affect the final size of the resulting significant networks. The statistical differences in demographic and clinical characteristics between PD patients and HC as well as between PD patients at different timepoints were computed using the t-test and the fisher’s test. The Wilcoxon test (two-tailed) corrected for multiple comparisons (Bonferroni) was used to examine the difference in the network between different visits.

### Correlation analysis

The Pearson’s correlation was computed to evaluate the relationship between the Network Index (NI) and the global clinical score of PD patients represented by the Montreal Cognitive Assessment score (MoCA)^1^ at different timepoints. Further, in order to emphasize the disrupted connections that reflect the fluctuations in the MoCA score over time, we sought also to identify a subnetwork from the significant network already uncovered in NBS, in which the change in the connectivity values of its edges correlates significantly with the change in the MoCA. For this purpose and inspired by the edges selection approach used in the Connectome-based Predictive modeling (CPM) studies^2^, the Pearson’s correlation was computed between the difference in the weight of the connections ($W_{i}^{BL}$ - $W_{i}^{5YFU}$) and the difference in the MoCA score (${MoCA}_{i}^{BL}$ - ${MoCA}_{i}^{5YFU}$) in both BL and 5YFU visits. Connections that show significant correlation (*p*<0.05) were considered as part of this subnetwork and their corresponding NI was computed.

We estimated also the correlation between the longitudinal change of NI and the longitudinal change in the UPDRS-III to assess the relationship between the loss of connectivity in PD patients and their motor impairments. The dose of antiparkinsonian medications (LEDD) was considered as a confounding factor when estimating the Pearson’s correlation at each timepoint (and its average between timepoints when correlating the longitudinal changes).

**Results**

#### Decreasing networks of PD patients in alpha1 and theta bands (BL>5YFU)

The alpha1 band (8-10 Hz) showed similar results to those observed in alpha2 concerning the predominance of connections in the right hemisphere as well as the significant progressive decreasing trend of NI between different visits in PD patients and not in HC (figure S2 A, B).

Another decreasing network was observed in the theta band (*t=3.2, p=0.036,* corrected using permutation) involving 83 connections and 65 regions distributed quasi-equally between both hemispheres. The highest degree regions were parts of the left-temporal, left-central and right-parietal lobes (figure S2 C). The NI in this band decreases significantly between BL and 5YFU (*p<0.001*) notably between 3YFU and 5YFU (*p<0.05*). However, this decreasing trend was also observed in HC with a significant difference between BL and 3YFU (*p<0.05*) (figure S2 D). We should note that results were consistent across different thresholds of the t-test in NBS, in all frequency bands (see table S2).

#### Increasing networks of PD patients (BL<5YFU)

Concerning the network where the connectivity in 5YFU was significantly higher than in BL (a hyper-connectivity), the alpha2 was the only frequency band to reveal significant components in NBS. The network (*t=3.1, p=0.025,* corrected using permutation) comprises 93 connections and 64 regions. Highest degree regions were among the right-parietal, right-frontal, right-occipital and left-parietal lobes and a predominance in the interhemispheric connections was observed (73.1%) (figure S2 E). These results are in line with the findings of Stoffers et al. that reported a positive association between the connectivity of the interhemispheric connections and the duration of the disease in alpha2^3^. However, we found that the associated NI goes through a progressive increase between BL and 5YFU (*p<0.001*) not only in PD patients, but also in HC between BL and 3YFU (*p<0.05*) (figure S2 F). This increase may be interpreted as age-related and not pathology- specific. However, this was not confirmed statistically as the correlation between the NI and the age was unsignificant (*p=0.62*).

#### Cross-validating the results on the entire cohort

In order to cross-validate the hypoconnectivity associated with the disrupted networks shown previously, we computed the corresponding NI on the initial cohort that includes in addition to the 35 PD patients of the analysis, the PD patients who did not complete the follow up-visits (total of 77 PD patients at BL, 45 PD at 3YFU and 42 PD at 5YFU).We chose to apply this validation on the decreasing networks of the alpha2 and beta bands as their corresponding NI showed a significant decrease in PD and not in HC. Results revealed that in alpha2, the significant progressive decreasing trend of NI persists on this larger dataset between BL and 3YFU (*p<0.05*) and BL and 5YFU (*p<0.001*). The same decreasing trend was also observed in beta mainly between BL and 5YFU (*p<0.001*) and 3YFU and 5YFU (*p<0.05*). Results are represented in the figure S3.

#### Relationship between the longitudinal change in the network and in the UPDRS-III score

In order to assess the relationship between the loss of the connectivity and the motor impairment in PD patients, we evaluated the correlation between the change in their NI and the change in their UPDRS-III score between different timepoints. A significant negative correlation was found in both frequency bands alpha2 (*r=-0.38, p<0.05*) and beta (*r=-0.41, p<0.05*) only between 3YFU and 5YFU (figure S5). This negative correlation reflects a worsening of motor symptoms associated with the loss of connectivity when the disease progress in time.

### Disrupted networks of PD patients with lateralized motor symptoms in beta band.

The dominance of the right hemisphere was observed in the detected dysconnectivity network of the beta band in LPD patients (*t=3.2, p=0.045*, *corrected*) that included 69.4% of the detected connections and 67.2% of the detected regions. The regions with the highest number of connections were part of the right-frontal, right-occipital and right-central lobes. The fronto-temporal and fronto-frontal were among the highest altered connections. the decrease in the NI was significant between BL and 5YFU (*p*<0.001) in LPD patients and not in RPD patients (figure 4 B) nor in HC between BL and 3YFU (*p=0.26*).

As for the RPD patients, the beta band revealed a network (*t=3.5, p=0.035,* corrected using permutation) with a confirmed dominance of the left hemisphere which comprises 86.1% of the total connections and 81.8% of the involved regions. The fronto-temporal and the fronto-parietal were the prominent altered interactions and the highest degree regions were among the frontal, central and occipital lobes of the left hemisphere. A progressive significant decrease in the corresponding NI was perceived in RPD patients between different visits: BL and 5YFU visits (*p<0.001*), BL and 3YFU (*p<0.05*), 3YFU and 5YFU (*p<0.05*). However, we observed this decrease also in the NI of the LPD patients between BL and 5YFU (*p<0.05*) (figure 4 D) while the change in HC between BL and 3YFU was not significant (*p*=0.07). All of these results were consistent independently from the chosen t-threshold for the t-test in NBS (see table S5).

**Supplementary figures**


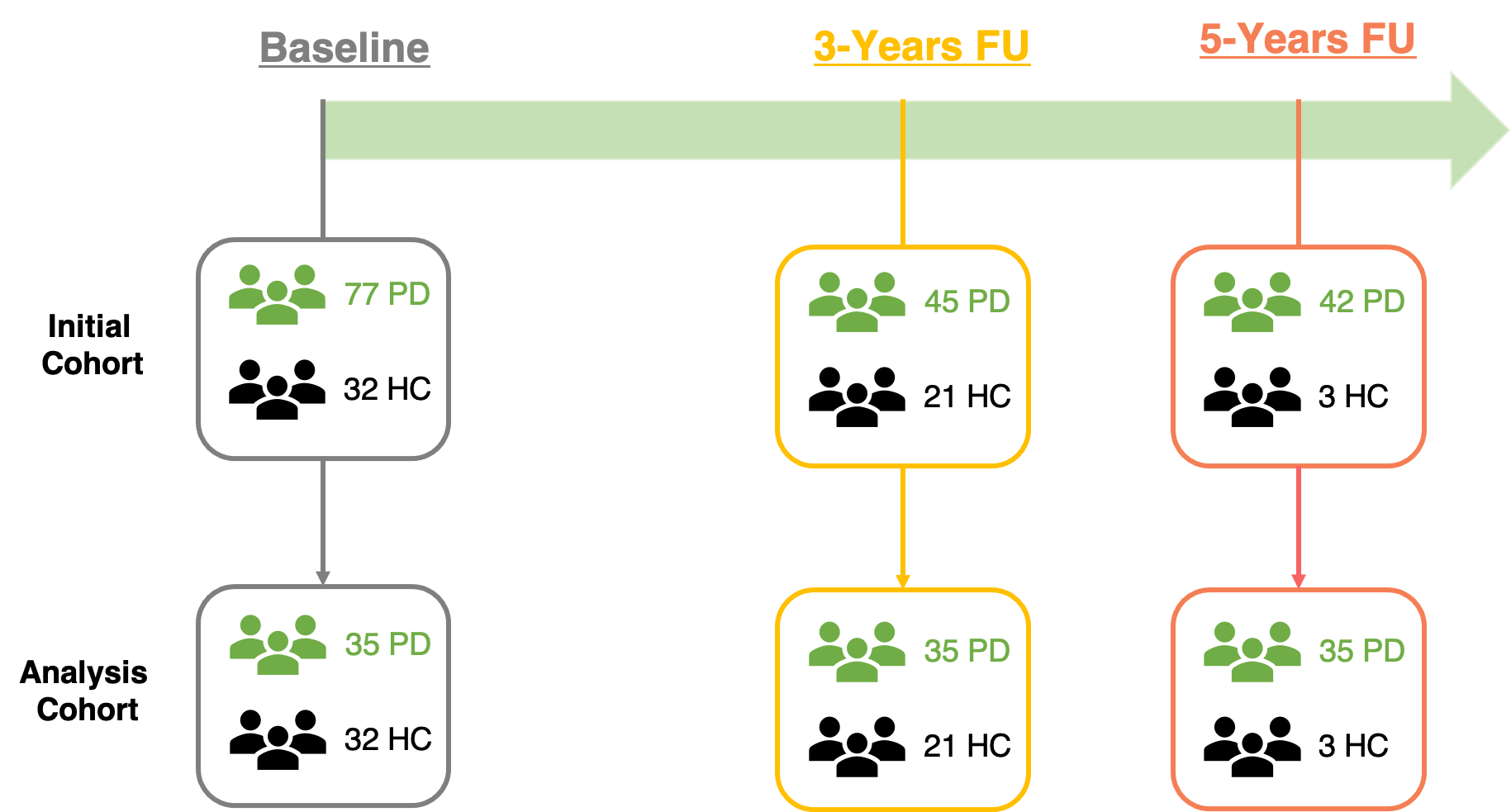


*Figure S1-Flowchart of the enrolled and followed subjects with Parkinson’s disease (PD) and healthy controls (HC) during the three timepoints of the study. The analysis cohort included only the 35 patients that underwent all three visits and all the HC. Seven patients from the 5YFU dataset were discarded from the analysis cohort as they were DBS patients at BL or at 3YFU (or at both timepoints) with no EEG recordings at that time.*


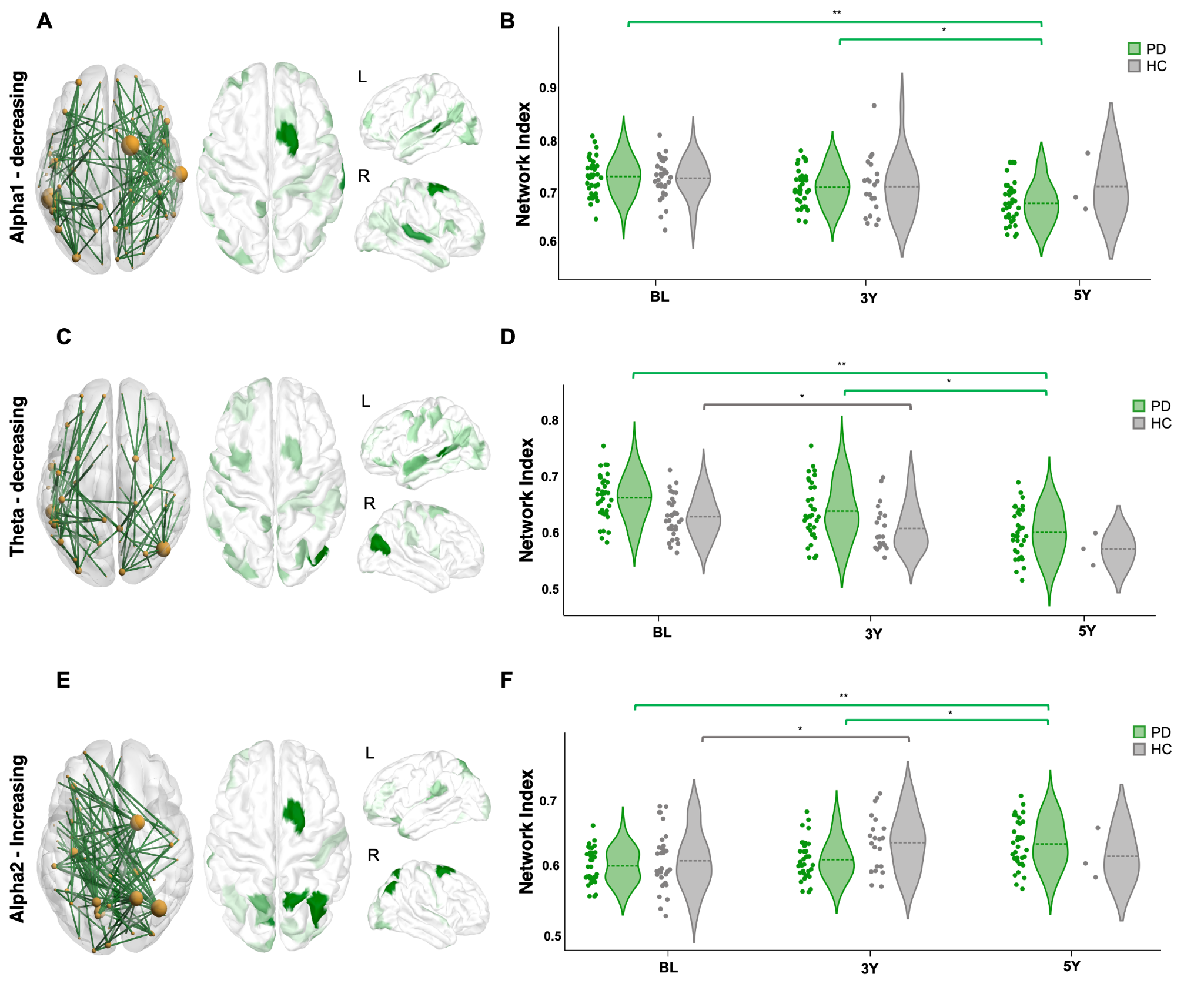


*Figure S2- Disrupted networks with corresponding highest degree regions in* ***A)*** *alpha1-decreasing* ***C)*** *theta-decreasing* ***E)*** *alpha2-increasing. The longitudinal change of the network index of the PD patients and HC in* ***B)*** *alpha1-decreasing* ***D)*** *theta-decreasing* ***F)*** *alpha2-increasing.
** p<0.001, * p<0.05 (corrected for multiple comparisons using Bonferroni).*


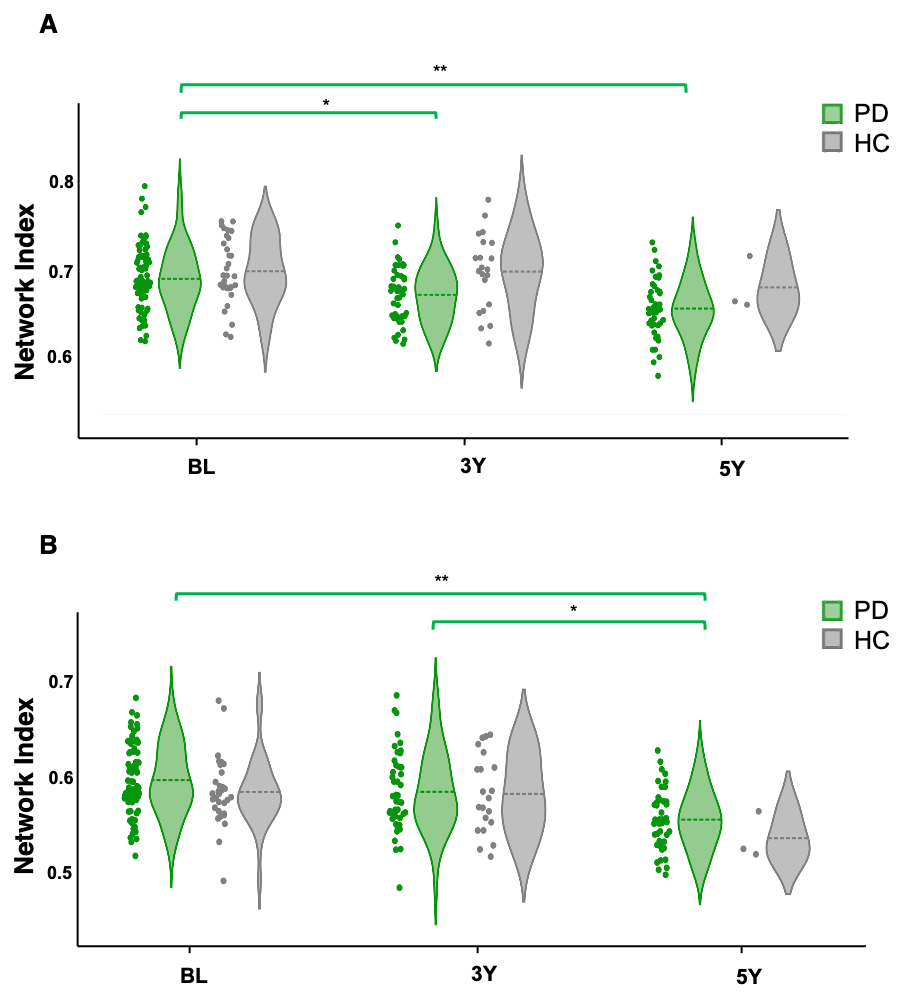


*Figure S3-Cross validation on the main cohort: the longitudinal change in the NI of PD patients and HC corresponding to the decreasing network of alpha2 (up) and the decreasing network of beta (down).
** p<0.001, * p<0.05 (corrected for multiple comparisons using Bonferroni)*


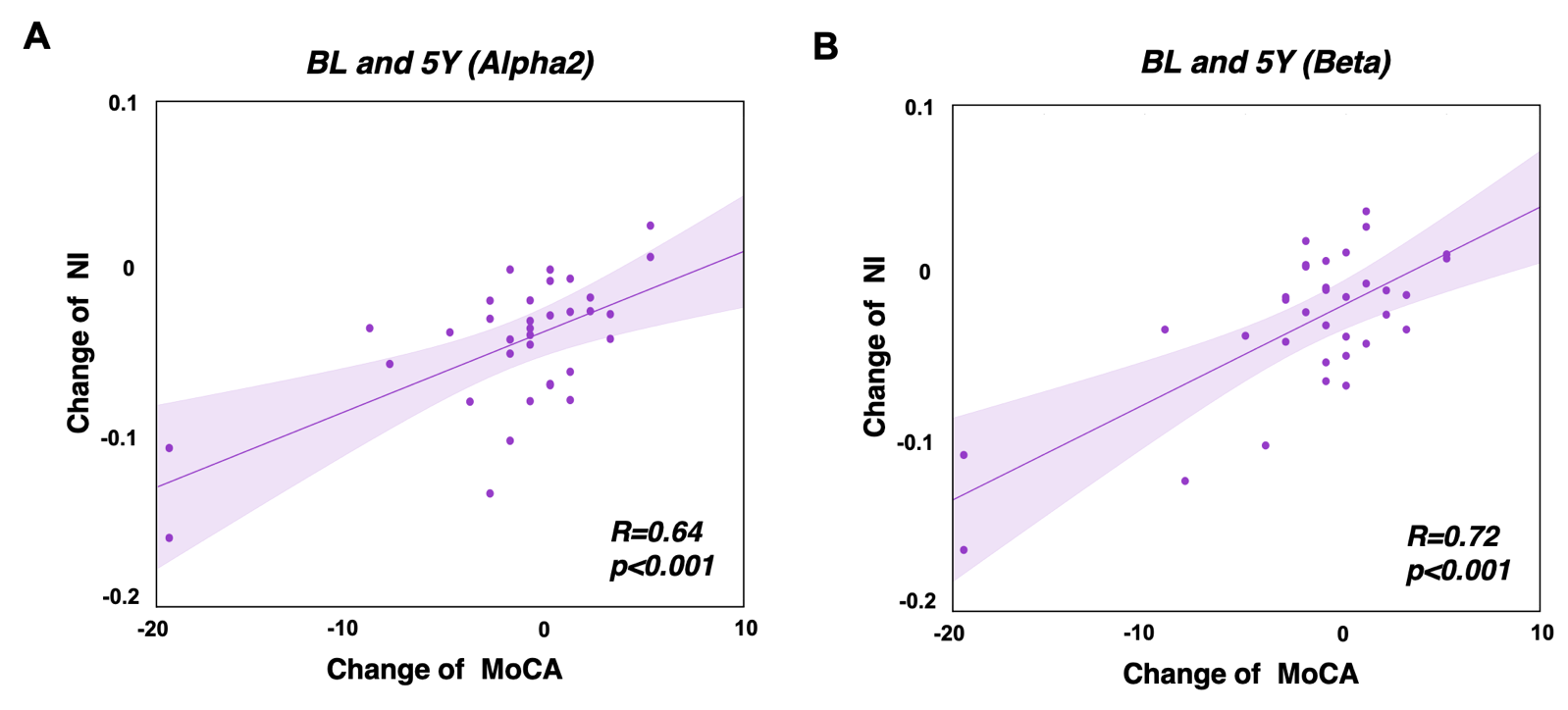


*Figure S4-Correlation between the change in Network Index (NI) and the change in MoCA observed between BL and 5YFU in* ***A****) Alpha2 and* ***B)*** *Beta.
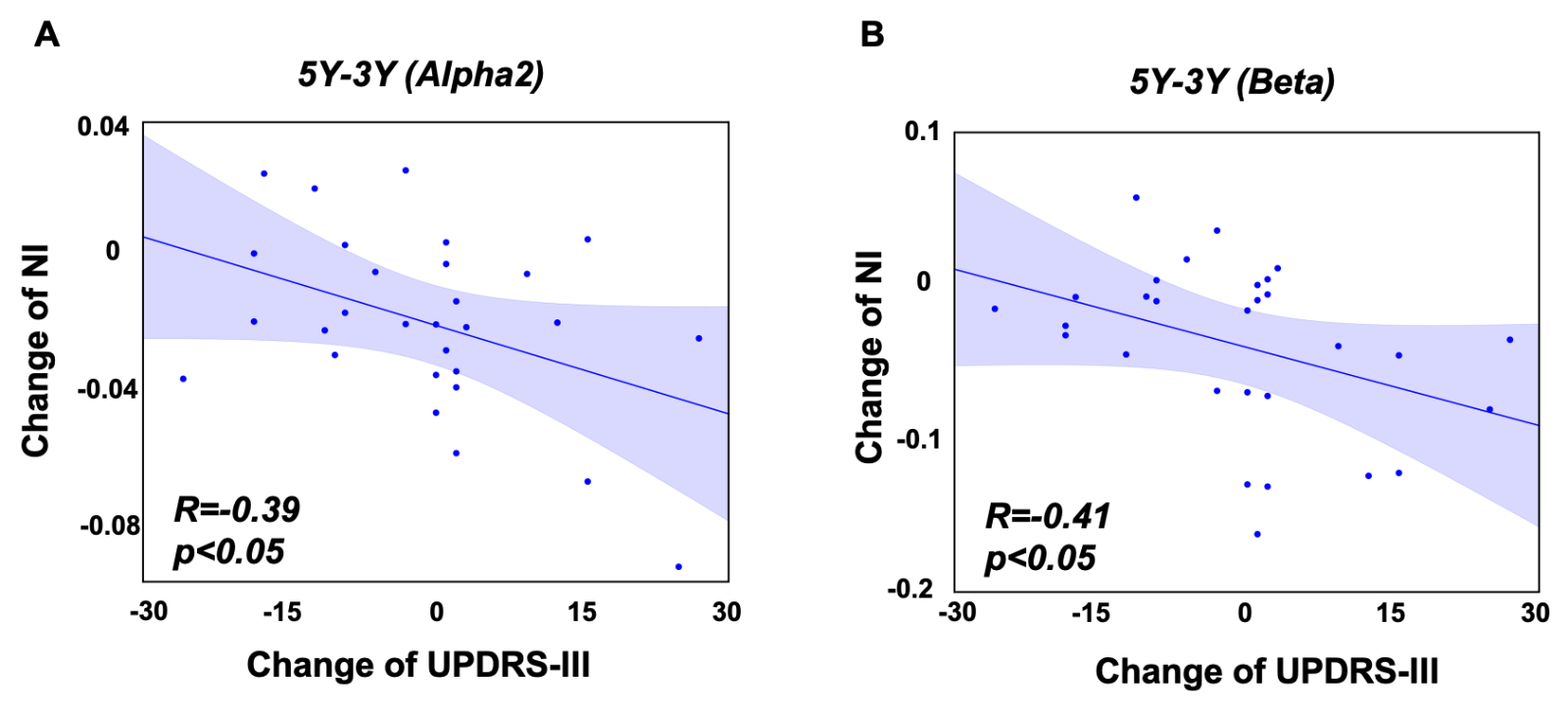
*

*Figure S5- Correlation between the change in Network Index (NI) and the change in UPDRS-III score observed between 3YFU and 5YFU in* ***A****) Alpha2 and* ***B)*** *Beta.*


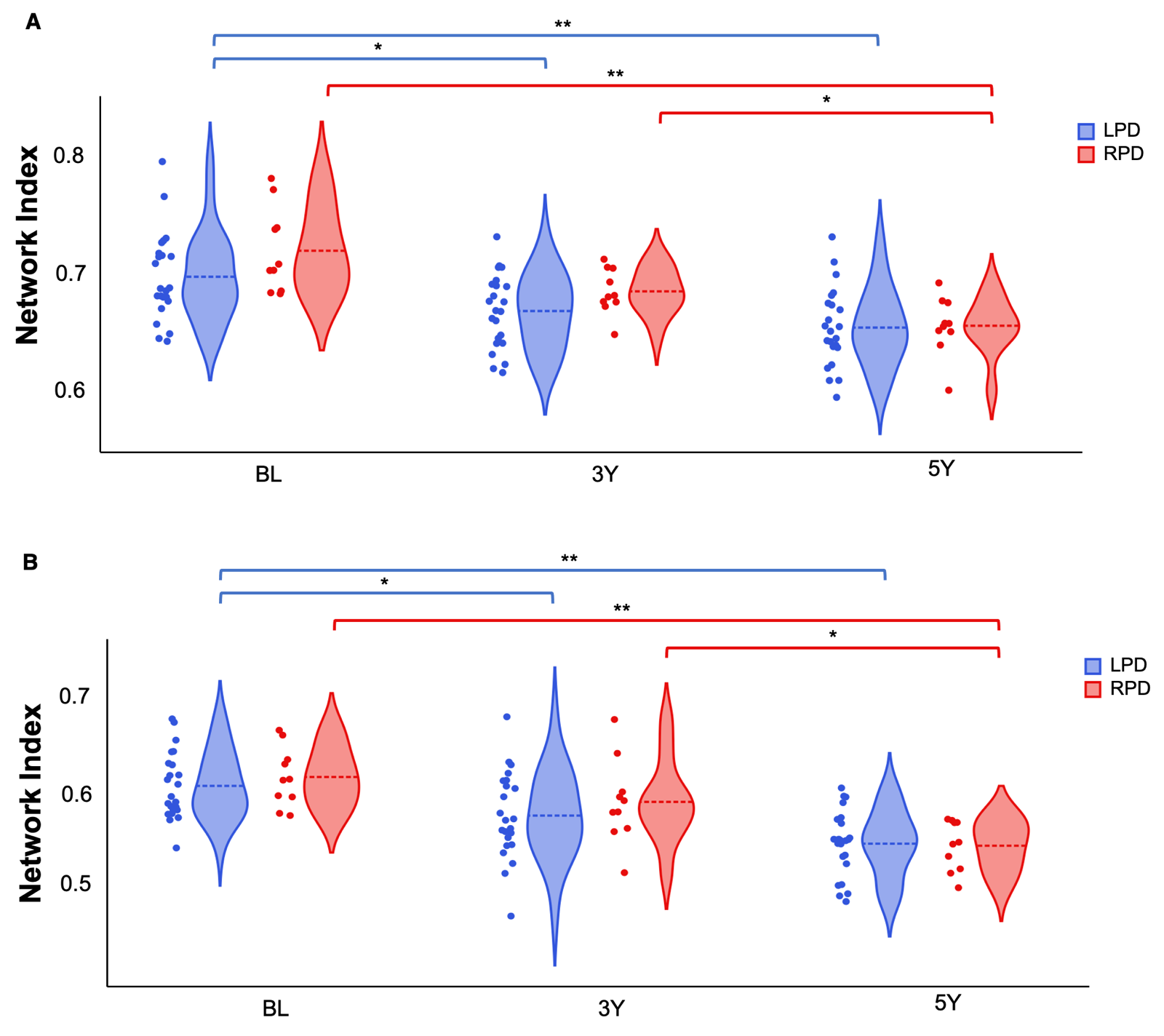


*Figure S6-The longitudinal change of the Network Index (NI) (derived from the dysconnectivity networks of all PD patients (N=35)) of the left-affected PD patients (LPD, N=23) and the right-affected PD patients (RPD, N=10) separately in* ***A)*** *alpha2 and* ***B)*** *beta frequency bands.
** p<0.01, * p<0.05 (corrected for multiple comparisons using Bonferroni).*

**Supplementary tables**

*Table S1-Demographic and clinical data of the initial cohort in Baseline, 3YFU and 5YFU expressed as mean (standard deviation). PD: Parkinson’s disease patients; HC: Healthy Controls; M/F: Male/Female; y: years; LEDD: Levodopa equivalent daily dose; UPDRS-III: Unified Parkinson’s Disease Rating Scales motor ratings; MoCA: Montreal Cognitive Assessment, MMSE: Mini-Mental State Examinations.*

|  | Baseline |  | 3 Years Follow UP |  | 5 Years Follow UP |
| --- | --- | --- | --- | --- | --- |
|  | PD (N=77) |  | PD (N=45) |  | PD (N=42) |
| Sex (M/F) | 51/26 |  | 31/14 |  | 28/14 |
| Age (y) | 66.2 (8.2) |  | 70.9 (7.9) |  | 71.9 (7.9) |
| Education (y) | 14.6 (3.2) |  | 14.8 (3.1) |  | 15.0 (3.1) |
| Disease Duration (y) | 5.4 (5.2) |  | 8.0 (5.2) |  | 10.5 (5.0) |
| LEDD (mg/day) | 676 (466) |  | 707 (447) |  | 642 (386) |
| UPDRS-III | 15.5 (11.0) |  | 20.5 (12.1) |  | 20.1 (13.1) |
| MoCA | 26 (2.4) |  | 25.2 (3.5) |  | 24.9 (5.2) |

*Table S2-Performance at the neuropsychological and neuropsychiatric tests of the 35 PD patient in the three visits: Baseline (BL), 3 years follow-up (3YFU) and 5 years follow-up (5YFU), expressed as mean (standard deviation).*

| Domain | Name of the test | BL | 3YFU | 5YFU |
| --- | --- | --- | --- | --- |
| Attention and working memory | TAP-Alertness, reaction time without alerting sound (ms) | 295 (61) | 312 (69) | 318 (64) |
|  | TAP-Alertness, reaction time with alerting sound (ms) | 281 (47) | 300 (67) | 304 (55) |
|  | Trail Making Test, time for Part A | 47.9 (15.4) | 52.8 (28.9) | 59.7 (36.4) |
|  | Digit Span, correct forward | 7.3 (2.1) | 7.8 (2.1) | 7.5 (1.7) |
|  | Digit span, correct backward | 5.9 (2.1) | 6.1 (1.7) | 5.7 (1.7) |
|  | Corsi block, correct forward | 7.9 (1.4) | 7.9 (1.7) | 7.5 (1.9) |
|  | Corsi block, correct backward | 7.5 (1.3) | 7.1 (1.7) | 5.9 (1.7) |
| Episodic Memory | California Verbal Learning Test, trial 1 | 5.5 (2) | 4.7 (2.4) | 4.9 (1.7) |
|  | California Verbal Learning Test, trial 5 | 10.9 (3) | 11 (3.4) | 10.4 (3.4) |
|  | California Verbal Learning Test, saving | 94.8 (21.2) | 82.4 (24.3) | 88.4 (24.8) |
|  | California Verbal Learning Test, discriminability | 98.8 (5.5) | 90.6 (8.7) | 90.8 (11.3) |
|  | Rey-Osterrieth Complex Figure, savings | 66.9 (15.5) | 70.6 (19.8) | 70 (20.2) |
| Executive Function | Five-Point test, correct answers | 24.7 (7.7) | 24.8 (7.1) | 25.6 (7) |
|  | Semantic verbal fluency test, correct answers | 20.2 (4.8) | 19.8 (6.5) | 19.3 (5.8) |
|  | Phonemic verbal fluency, correct answers | 13.2 (4.1) | 13 (4.9) | 13.7 (4.9) |
|  | Trail Making Test,time for Part B / time for Part A | 2.5 (0.8) | 2.6 (1.6) | 3 (1.6) |
|  | Stroop, interference index | 1.9 (0.6) | 1.8 (0.5) | 1.9 (0.7) |
| Visuo-spatial function | Rey-Osterrieth Complex Figure, copy | 29.9 (4) | 29.3 (5.6) | 30 (5.4) |
|  | Block design test, sum score | 26.4 (8.6) | 26.4 (8.8) | 24.5 (7.7) |
| Language | Boston Naming Test, correct answers | 14.1 (1) | 13.9 (1.7) | 13.8 (2.6) |
| Neuropsychiatry | Beck Depression Inventory, total | 7.6 (5) | 8.2 (5) | 7.5 (6.8) |
|  | Beck Anxiety Inventory, total | 10.5 (7.1) | 11.9 (8) | 10.2 (7.4) |
|  | Perceived deficits questionnaire - depression, total | 32.7 (21.1) | 37.4 (22.2) | 36 (20) |

*Table S3-Demographic and clinical characteristics of the Left-affected (LPD) and right-affected (RPD) patients in BL, 3YFU and 5YFU* ex*pressed as Median [min, max]. M/F: Male/Female; y: years; LEDD: Levodopa equivalent daily dose; UPDRS-III: Unified Parkinson’s Disease Rating Scales motor ratings; MoCA: Montreal Cognitive Assessment (score between 0 and 30). P-values between groups are calculated using the Wilcoxon’s statistical test.*

|  | Baseline | |  | | 3 Years Follow UP | |  | | 5 Years Follow UP | |  | |
| --- | --- | --- | --- | --- | --- | --- | --- | --- | --- | --- | --- | --- |
|  | LPD  (N=23) | RPD  (N=10) | | *p-value* | LPD  (N=23) | RPD  (N=10) | | *p-value* | LPD  (N=23) | RPD  (N=10) | | *p-value* |
| Sex (M/F) | 19M/4F | 5M/5F | | *0.09* | - | - | | *-* | - | - | | - |
| Age (y) | 69 [47-82] | 65.5 [55-84] | | *0.46* | 72 [50-85] | 68.5 [58-87] | | *0.43* | 74 [52-87] | 70.5 [60-89] | | 0.44 |
| Education (y) | 16 [9-20] | 13.5 [9-20] | | *0.19* | - | - | | *-* | - | - | | - |
| Disease Duration (y) | 2.8 [0-15.3] | 4.2 [0.1-8.2] | | *0.54* | 5.9 [3.1-18.5] | 7.25 [3.5-11.8] | | *0.57* | 8.1 [5.2-20.5] | 9.3 [5.5-13.3] | | 0.57 |
| LEDD (mg/day) | 460 [0-1425] | 575 [150-1148] | | *0.31* | 512.5 [0-2028] | 585 [114-1480] | | *0.85* | 574.5 [0-1330] | 537 [94-1629] | | 0.96 |
| UPDRS-III | 16 [0-34] | 11.5 [1-35] | | *0.37* | 17 [1-34] | 18.5 [10-38] | | *0.48* | 15 [1-52] | 11 [0-37] | | 0.38 |
| MoCA | 27 [22-29] | 25 [19-29] | | *0.1* | 26 [15-29] | 26 [12-30] | | *0.96* | 27 [4-29] | 26 [17-30] | | 0.94 |

*Table S4-Results using different t-test thresholds in NBS when finding the hypo/hyperconnectivity networks between BL and 5YFU of the PD patients in different frequency bands. t-thresh: the t-test threshold used in NBS, p-value: the corrected p-value using NBS, N° Edges: the number of edges in the significant network, N° Regions: the number of regions in significant network, Edges within RH: the percentage of edges within the right hemisphere, NI-PD/HC (p-value): the significance of the Wilcoxon test (corrected for multiple comparison using Bonferroni) computed to evaluate the difference between the network index of PD patients between different timepoints and of Healthy controls between BL and 3YFU.
Significant p-values are corrected for multiple comparisons using Bonferroni.
* p-value<0.05 without correction for multiple comparison.*

|  |  |  |  |  |  | NI-PD (p-value) | | | NI-HC (p-value) |
| --- | --- | --- | --- | --- | --- | --- | --- | --- | --- |
|  | t-thresh | p-value | N° Edges | N° Regions | Edges in RH | BL - 5Y | BL - 3Y | 3Y - 5Y | BL - 3Y |
| Alpha 2 hypo-Con | 2.6 | 0.050 | 466 | 151 | 69% | <0.001 | <0.01 | <0.05 | 0.86 |
|  | 2.9 | 0.034 | 275 | 128 | 73% | <0.001 | <0.01 | <0.05 | 0.87 |
|  | 3.0 | 0.033 | 209 | 109 | 78% | <0.001 | <0.01 | <0.05 | 0.89 |
|  | 3.1 | 0.033 | 182 | 104 | 78% | <0.001 | <0.01 | <0.05 | 0.86 |
|  | 3.2 | 0.021 | 125 | 72 | 89% | <0.001 | <0.01 | <0.05 | 0.86 |
|  | 3.5 | 0.028 | 29 | 27 | 93% | <0.001 | <0.01 | <0.05 | 0.69 |
|  | 3.7 | 0.023 | 20 | 18 | 90% | <0.001 | <0.01 | <0.05 | 0.24 |
| Beta hypo-Con | 2.6 | 0.038 | 817 | 158 | 39% | <0.001 | 0.031* | <0.001 | 0.69 |
|  | 2.9 | 0.025 | 500 | 139 | 41% | <0.001 | 0.017* | <0.001 | 0.67 |
|  | 3.0 | 0.022 | 420 | 130 | 43% | <0.001 | <0.05 | <0.001 | 0.64 |
|  | 3.1 | 0.019 | 359 | 123 | 45% | <0.001 | <0.05 | <0.001 | 0.61 |
|  | 3.2 | 0.018 | 302 | 118 | 46% | <0.001 | <0.05 | <0.001 | 0.61 |
|  | 3.5 | 0.011 | 158 | 76 | 55% | <0.001 | <0.05 | <0.001 | 0.92 |
|  | 3.7 | 0.009 | 103 | 58 | 56% | <0.001 | <0.05 | <0.001 | 0.75 |
| Theta hypo-Con | 2.6 | - | - | - | - | - | - | - | - |
|  | 2.9 | - | - | - | - | - | - | - | - |
|  | 3.0 | - | - | - | - | - | - | - | - |
|  | 3.1 | 0.045 | 104 | 73 | - | <0.001 | 0.034* | <0.05 | 0.05 |
|  | 3.2 | 0.036 | 83 | 65 | - | <0.001 | 0.028* | <0.05 | 0.037 |
|  | 3.5 | 0.041 | 17 | 18 | - | <0.001 | 0.135 | <0.01 | 0.035 |
|  | 3.7 | - | - | - | - | - | - | - | - |
| Alpha1 hypo-Con | 2.6 | 0.044 | 488 | 161 | 51% | <0.001 | 0.038* | <0.05 | 0.21 |
|  | 2.9 | 0.038 | 248 | 118 | 51% | <0.001 | 0.024* | <0.01 | 0.13 |
|  | 3.0 | 0.035 | 195 | 105 | 53% | <0.001 | 0.022* | <0.01 | 0.13 |
|  | 3.1 | 0.033 | 149 | 93 | 51% | <0.001 | 0.02* | <0.01 | 0.14 |
|  | 3.2 | 0.031 | 121 | 81 | 47% | <0.001 | 0.019* | <0.01 | 0.09 |
|  | 3.5 | 0.020 | 52 | 49 | 50% | <0.001 | 0.018* | <0.01 | 0.06 |
|  | 3.7 | 0.023 | 18 | 19 | 55% | <0.001 | <0.05 | <0.001 | 0.26 |
| Alpha2 hyper-Con | 2.6 | 0.049 | 334 | 105 | - | <0.01 | 0.23 | <0.05 | 0.025 |
|  | 2.9 | 0.035 | 168 | 79 | - | <0.01 | 0.12 | <0.05 | 0.023 |
|  | 3.0 | 0.028 | 139 | 75 | - | <0.01 | 0.14 | <0.05 | 0.023 |
|  | 3.1 | 0.026 | 106 | 66 | - | <0.01 | 0.13 | <0.05 | 0.023 |
|  | 3.2 | 0.023 | 75 | 55 | - | <0.01 | 0.18 | <0.05 | 0.016 |
|  | 3.5 | 0.015 | 20 | 21 | - | <0.01 | 0.32 | <0.05 | 0.015 |
|  | 3.7 | - | - | - | - | - | - | - | - |

*Table S5-Results using different t-test thresholds in NBS when finding the hypo-connectivity networks between BL and 5YFU of the PD patients in different frequency bands. t-thresh: the t-test threshold used in NBS, p-value: the corrected p-value using NBS, N° Edges: the number of edges in the significant network, N° Regions: the number of regions in significant network, Edges within LH/RH: the percentage of edges within the left/right hemisphere, NI (p-value): the significance of the Wilcoxon test computed to evaluate the difference between the network index of LPD or RPD patients between different timepoints.
Significant p-values are corrected for multiple comparisons using Bonferroni.
* p-value<0.05 without correction for multiple comparison.*

|  |  |  |  |  |  |  | NI (p-value) | | |
| --- | --- | --- | --- | --- | --- | --- | --- | --- | --- |
|  | t-thresh | p-value | N° Edges | N° Regions | Edges in LH | Edges in RH | BL - 5Y | BL - 3Y | 3Y - 5Y |
| LPD Alpha2 | 2.7 | - | - | - | - | - | - | - | - |
|  | 2.9 | 0.045 | 103 | 71 | 9% | 60% | <0.001 | 0.027* | <0.05 |
|  | 3.0 | 0.039 | 81 | 62 | 6% | 63% | <0.001 | 0.033* | <0.05 |
|  | 3.1 | 0.038 | 53 | 46 | 8% | 66% | <0.001 | 0.028* | <0.05 |
|  | 3.2 | 0.038 | 28 | 24 | 0% | 89% | <0.001 | 0.023* | <0.05 |
|  | 3.5 | 0.039 | 5 | 6 | 0% | 100% | <0.05 | 0.048* | 0.071 |
|  | 3.7 | - | - | - | - | - | - | - | - |
| LPD  Beta | 2.7 | - | - | - | - | - | - | - | - |
|  | 2.9 | - | - | - | - | - | - | - | - |
|  | 3.0 | 0.046 | 217 | 57 | 6% | 67% | <0.001 | 0.061 | <0.05 |
|  | 3.1 | 0.049 | 181 | 67 | 6% | 70% | <0.001 | 0.058 | 0.022* |
|  | 3.2 | 0.045 | 147 | 61 | 5% | 69% | <0.001 | 0.048* | 0.025* |
|  | 3.5 | 0.025 | 88 | 44 | 1% | 80% | <0.001 | 0.031* | 0.028* |
|  | 3.7 | 0.017 | 59 | 32 | 2% | 83% | <0.001 | 0.029* | 0.026* |
| RPD Alpha2 | 2.7 | 0.046 | 501 | 164 | 47% | 42% | <0.001 | <0.05 | <0.05 |
|  | 2.9 | 0.042 | 301 | 114 | 54% | 36% | <0.01 | <0.05 | 0.017* |
|  | 3.0 | 0.049 | 262 | 107 | 55% | 37% | <0.001 | <0.05 | <0.05 |
|  | 3.1 | 0.035 | 215 | 100 | 53% | 38% | <0.01 | <0.05 | 0.017* |
|  | 3.2 | 0.044 | 167 | 83 | 53% | 40% | <0.001 | <0.01 | 0.017* |
|  | 3.5 | 0.029 | 94 | 56 | 52% | 44% | <0.01 | <0.05 | <0.05 |
|  | 3.7 | 0.022 | - | - | 51% | 45% | <0.001 | <0.05 | <0.05 |
| RPD  Beta | 2.7 | 0.045 | 686 | 174 | 66% | 16% | <0.001 | <0.05 | <0.05 |
|  | 2.9 | 0.040 | 520 | 159 | 70% | 14% | <0.001 | <0.05 | <0.05 |
|  | 3.0 | 0.048 | 445 | 149 | 73% | 13% | <0.001 | <0.05 | <0.05 |
|  | 3.1 | 0.037 | 387 | 141 | 75% | 11% | <0.001 | <0.05 | <0.05 |
|  | 3.2 | 0.035 | 323 | 131 | 75% | 11% | <0.001 | <0.05 | <0.05 |
|  | 3.5 | 0.035 | 194 | 88 | 86% | 5% | <0.001 | <0.05 | <0.05 |
|  | 3.7 | 0.028 | 113 | 48 | 97% | 0% | <0.001 | <0.05 | 0.037* |

**References**

1. Nasreddine ZS, Phillips NA, Bédirian V, et al. The Montreal Cognitive Assessment, MoCA: A Brief Screening Tool For Mild Cognitive Impairment. *J Am Geriatr Soc*. 2005;53(4):695-699. doi:10.1111/j.1532-5415.2005.53221.x

2. Shen X, Finn ES, Scheinost D, et al. Using connectome-based predictive modeling to predict individual behavior from brain connectivity. *Nat Protoc*. 2017;12(3):506-518. doi:10.1038/nprot.2016.178

3. Stoffers D, Bosboom JLW, Deijen JB, Wolters ECh, Stam CJ, Berendse HW. Increased cortico-cortical functional connectivity in early-stage Parkinson’s disease: An MEG study. *NeuroImage*. 2008;41(2):212-222. doi:10.1016/j.neuroimage.2008.02.027
